# AGO1 and AGO2 Act Redundantly in miR408-Mediated *Plantacyanin* Regulation

**DOI:** 10.1371/journal.pone.0028729

**Published:** 2011-12-13

**Authors:** Nicolas Maunoury, Hervé Vaucheret

**Affiliations:** Institut Jean-Pierre Bourgin, INRA, Versailles, France; Ecole Normale Superieure, France

## Abstract

**Background:**

In Arabidopsis, AGO1 and AGO2 associate with small RNAs that exhibit a Uridine and an Adenosine at their 5′ end, respectively. Because most plant miRNAs have a 5′U, AGO1 plays many essential roles in miRNA-mediated regulation of development and stress responses. In contrast, AGO2 has only been implicated in antibacterial defense in association with miR393*, which has a 5′A. AGO2 also participates in antiviral defense in association with viral siRNAs.

**Principal Findings:**

This study reveals that miR408, which has a 5′A, regulates its target *Plantacyanin* through either AGO1 or AGO2. Indeed, neither *ago1* nor *ago2* single mutations abolish miR408-mediated regulation of *Plantacyanin*. Only an *ago1 ago2* double mutant appears compromised in miR408-mediated regulation of *Plantacyanin*, suggesting that AGO1 and AGO2 have redundant roles in this regulation. Moreover, the nature of the 5′ nucleotide of miR408 does not appear essential for its regulatory role because both a wildtype *5′A-MIR408* and a mutant *5′U-MIR408* gene complement a *mir408* mutant.

**Conclusions/Significance:**

These results suggest that miR408 associates with both AGO1 and AGO2 based on criteria that differ from the 5′ end rule, reminiscent of miR390-AGO7 and miR165/166-AGO10 associations, which are not based on the nature of the 5′ nucleotide.

## Introduction

MicroRNAs are 20–24 nucleotides (nt) long riboregulators involved in many developmental processes and stress responses, acting through mRNA cleavage or translational repression [1], [2], [3], [4]. miRNAs have been found in plants and animals. MIR genes are translated by RNA polymerase II. The resulting single-stranded transcripts fold into imperfect hairpins that are processed by Dicer enzymes into mature miRNA that are loaded onto Argonaute proteins, which execute silencing activity. Most genomes also encode siRNAs either by transcription of long-inverted repeats, transcription of genes organized in convergent orientation, or transformation of single-stranded RNA into double-stranded RNA (dsRNA) by RNA-dependent RNA polymerase [2], [5]. Similar to miRNAs, siRNAs are processed by Dicer enzymes and loaded onto Argonaute proteins. Plants and invertebrates also produce siRNA from exogenously introduced RNA (transgenes, viruses, bacteria, etc). These siRNA target the RNA they originate from, providing an immune response [3], [6].

The plant model species Arabidopsis thaliana encode ten Argonaute (AGO) proteins [3]. Analysis of small RNA cloned after specific AGO pull-down revealed distinct features for different AGO proteins [7], [8], [9], [10]. AGO4, AGO6 and AGO9 proteins mostly associate with 24-nt siRNA, whereas AGO1, AGO2, AGO5, AGO7 and AGO10 mostly associate with 21–22-nt molecules. AGO7 and AGO10 show an almost exclusive association with miR390 and miR165/166, respectively, whereas AGO1, AGO2 and AGO5 associate with small RNA that exhibit a Uridine, an Adenosine and a Cytosine at their 5′ end, respectively. Because most plant miRNAs start with a Uridine, *ago1* null alleles exhibit pleiotropic developmental defects. Null alleles impaired in other AGO members exhibit either limited developmental defects (*ago7*, *ago10*) or no developmental defects when grown under standard conditions in the laboratory (*ago2*, *ago3*, *ago4*, *ago5*, *ago6*, *and ago9*) [9].

For a long time, no role has been assigned to AGO2. AGO2 and AGO3 differ from other plant AGO because they lack the canonical DDH motif in the cleavage catalytic site [11]. So far, evidence that Arabidopsis AGO2 could perform RNA cleavage is missing. Rather, AGO2 was proposed to prevent miRNA action because mutagenizing 5′U-miRNAs into 5′A-miRNAs redirected the miRNAs from AGO1 to AGO2 and altered their biological activity [7], [12]. Only recently, AGO2 was shown to play a regulatory role in association with a miRNA star molecule, i.e. the passenger strand of the miRNA duplex produced by Dicer. Indeed, miR393*, which has a 5′A, associates with AGO2, and *ago2* mutants are impaired in the regulation of genes involved in the response to bacterial infection [13]. AGO2 was also shown to participate in plant antiviral response [14], [15], [16], [17]. In the case of two viruses, AGO2 seem to cooperate with AGO1 to promote efficient antiviral activity [14], [17], whereas in another case, only AGO2, but not AGO1, seems to be involved in antiviral activity [15].

miR408 is one of the few miRNAs that exhibits an Adenosine at its 5′ end. Consistent with the AGO-miRNA 5′ end association rule, miR408 shows one of the highest enrichment in AGO2 pull-down experiments. miR408 accumulates at low levels in plants grown under standard conditions, but its accumulation is induced by various stresses, including copper starvation in Arabidopsis thaliana [18], [19] and drought stress in *medicago truncatula* [20]. miR408 exhibits extensive complementarity with three members of the Laccase family (*LAC3*, *LAC12*, *LAC13*) and with a gene encoding *Plantacyanin* (At2g02850). Cleavage of the four target mRNAs in the middle of the miR408 complementarity sequence was previously demonstrated [18], but the AGO protein responsible for this cleavage was not identified. Here we show that AGO1 and AGO2 likely act redundantly to regulate *Plantacyanin* mRNA levels. We also show that the presence of an Adenosine at the 5′ end of miR408 is not required for miR408-mediated *Plantacyanin* regulation.

## Results

### miR408-mediated Plantacyanin regulation is not impaired in ago2 mutants

**Figure 1:**
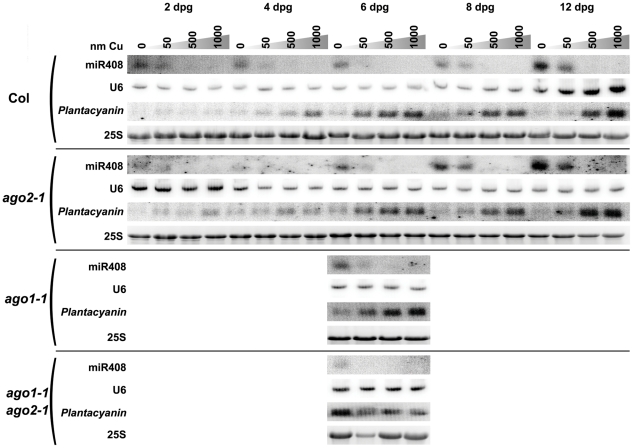
*ago1 ago2* double mutants but not *ago1* or *ago2* single mutants are compromised in miR408-mediated silencing of *Plantacyanin*. RNA gel blot analysis of miR408 and *Plantacyanin* in seedlings of wildtype Col (A), *ago2-1* null allele (B), *ago1-1* null allele (C) and *ago1-1 ago2-1* double mutant (D) grown in vitro in the absence of CuSO_4_ or on medium supplemented with 50, 500 or 1000 nm of CuSO_4_. Analysis of Col and *ago2-1* was performed 2, 4, 6, 8 and 10 days after germination (dag). Analysis of *ago1-1* and *ago1-1 ago2-1* was only performed at 6 dag. U6 and 25S hybridization were used as loading controls.

The complementarity between *Plantacyanin* and miR408, and the location of the complementarity site within the *Plantacyanin* mRNA are widely conserved among the plant kingdom, including plants as distant as wheat, grapevine, poplar and Arabidopsis, supporting a conserved role of miR408 in *Plantacyanin* regulation. miR408 exhibits an Adenosine at its 5′ end and is enriched in the AGO2-associated small RNA fraction, suggesting that it could regulate its target *Plantacyanin* through AGO2. To determine if AGO2 is required for miR408-mediated *Plantacyanin* regulation, we analyzed mature miR408 and *Plantacyanin* mRNA levels in wild-type plants and in the *ago2-1* null allele. The analysis was performed at different stages of development using plants grown in vitro under various concentrations of copper. Figure 1A shows that in wildtype plants the highest level of miR408 accumulation is observed in the absence of copper in the growth medium. *Plantacyanin* mRNA levels inversely correlate with miR408 levels, confirming that miR408 regulates *Plantacyanin* mRNA levels. No difference was observed between wild-type plants and the *ago2-1* null allele (Figure 1B), suggesting either that AGO2 is not required for miR408-mediated *Plantacyanin* regulation, or that another AGO can bind miR408 and fulfill regulatory activity in *ago2* mutants.

### AGO1 and AGO2 likely act redundantly to regulate Plantacyanin mRNA levels

To determine if AGO1 participates in miR408-mediated *Plantacyanin* regulation, we analyzed mature miR408 and *Plantacyanin* mRNA levels in the *ago1-1* null allele and in the *ago1-1 ago2-1* double mutant. The analysis was performed at a single stage of development using plants grown in vitro under the same range of copper concentration. The comparison of Figures 1A, 1B and 1C show no difference between wild-type plants and the *ago1-1* or *ago2-1* null alleles, suggesting either redundant roles for AGO1 and AGO2 or the involvement of yet another AGO protein in the regulation of *Plantacyanin* mRNA levels by miR408. Analysis of the *ago1-1 ago2-1* double mutant revealed a high level of *Plantacyanin* mRNA in copper-free conditions (Figure 1D), indicating that in the absence of AGO1 and AGO2, miR408 fails to properly regulate *Plantacyanin* mRNA levels. Although we cannot formally exclude that another AGO could contribute to the regulation of *Plantacyanin* mRNA levels by miR408, these results strongly suggest that AGO1 and AGO2 act redundantly in this regulation.

### miR408 associates with AGO1 and AGO2

The sequencing of small RNAs associated with AGO2 in flowers revealed a strong enrichment for miR408, miR852 and miR866 [8]. To determine the fraction of miR408 that associates with AGO1 and AGO2 in seedlings, which were analyzed in our experiments, we mined small RNA databases. In seedlings, only a comparison of total small RNAs and AGO1-associated small RNAs could be obtained [21]. As shown on Table 1, the 21-nt 5′A molecule that defines the canonical miR408 represents the most abundant miR408 form found in seedlings. miR408 variants include a 20-nt 5′A molecule, as well as 20-, 21- and 22-nt 5′U molecules, which start at position 2 of the canonical miR408. Surprisingly, a 22-nt 5′U molecule that differs at the 22^nd^ position is also found, which represents the second most abundant form of miR408. It is likely that it corresponds to an edited form of miR408 [22]. Altogether, 5′A miR408 molecules represent 68% of the miR408 molecules, while 5′U miR408 molecules represent 32%. In the AGO1-associated small RNA fraction, the 5′A miR408 molecules (including 20- and 21-nt forms) represent 37% of the miR408 molecules, while the 5′U miR408 molecules (including 20-, 21- and 22-nt, wildtype and edited forms) represent 63% (Table 1), indicating that 5′A miR408 does not exclusively associates with AGO2. If we assume that 100% of the 5′U miR408 molecules associate with AGO1, this result suggests that 27,6% of 5′A miR408 molecules associate with AGO1, and 72,4% with AGO2.

**Table 1:** Numbers of miR408 reads in total small RNA and AGO1-IP small RNA libraries prepared from Arabidopsis seedlings.

miR408 molecules in seedlings	Total	AGO1-IP
ATGCACTGCCTCTTCCCTGG	5	291
ATGCACTGCCTCTTCCCTGGC	575	2812
TGCACTGCCTCTTCCCTGGC	37	1131
TGCACTGCCTCTTCCCTGGCT	86	2149
TGCACTGCCTCTTCCCTGGCTC	20	247
TGCACTGCCTCTTCCCTGGCTT	118	1762
5′A miR408	68%	37%
5′T miR408	32%	63%

The fraction of 5′A and 5′U molécules in each library is indicated in percentage.

### 5′Adenosine is not required for miR408-mediated Plantacyanin regulation

Our results indicate that AGO1 and AGO2 likely act redundantly to regulate *Plantacyanin*. However, because 5′A miR408 molecules associate with both AGO1 and AGO2, it remains unclear if this dual regulation occurs exclusively through the canonical 21-nt 5′A miR408 molecule associated with both AGO1 and AGO2, and if this regulation requires the presence of an Adenosine at the 5′ end of miR408. To answer this question we asked whether a *5′U-MIR408* mutant gene in which the native 5′ Adenosine has been replaced by an Uridine could complement *mir408* mutants as efficiently as the *5′A-MIR408* wild-type gene.

**Figure 2:**
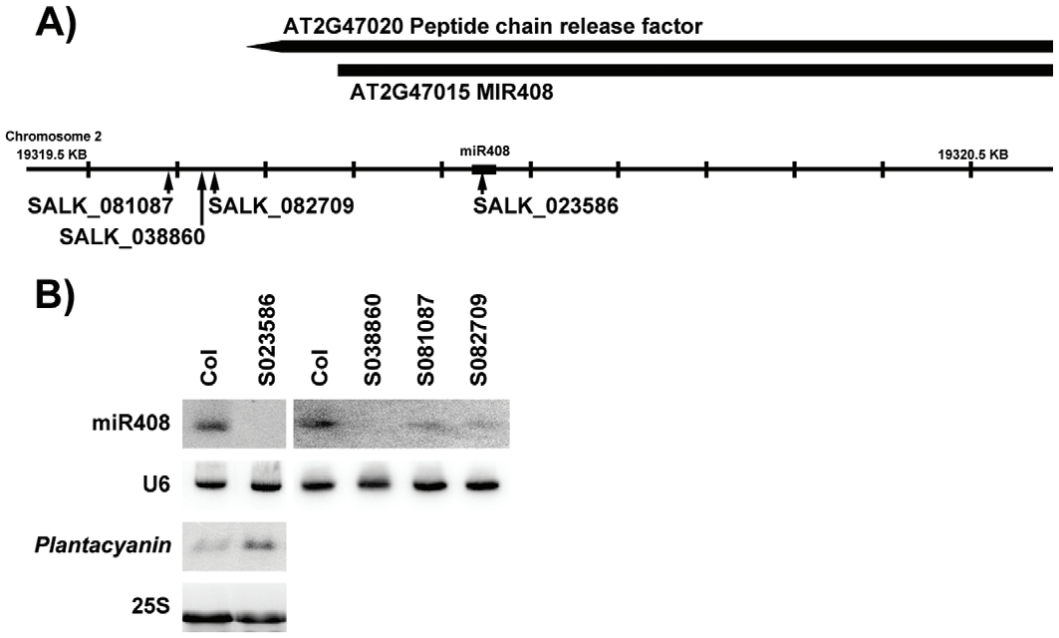
Identification of *mir408* null alleles. A) Location of the T-DNA inserted in the MIR408 gene in lines SALK_081087, SALK_082709, SALK_023586 and SALK_038860. B) RNA gel blot analysis of miR408 and *Plantacyanin* in seedlings of wildtype Col and *mir408* mutants. Plants were grown in vitro in the absence of CuSO_4_. U6 and 25S hybridization were used as loading controls.

**Figure 3:**
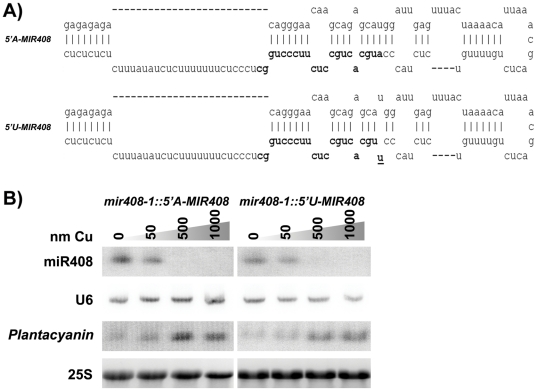
Complementation of a *mir408* null allele by the *5′A-MIR408* wild-type gene and a *5′U-MIR408* mutant gene. A) Sequence and structure of the MIR408 foldback stem loop in the *5′A-MIR408* wild-type gene and in the *5′U-MIR408* mutant gene. The mutagenized base is underlined. B) RNA gel blot analysis of miR408 and *Plantacyanin* in seedlings of *mir408* mutants transformed with either the *5′A-MIR408* wild-type gene or the *5′U-MIR408* mutant gene. Plants were grown in vitro in the absence of CuSO_4_ or on medium supplemented with 50, 500 or 1000 nm of CuSO_4_. U6 and 25S hybridization were used as loading controls.

Various Arabidopsis T-DNA lines carrying insertions in the *MIR408* (Figure 2A) were tested for miR408 accumulation. Lines SALK_081087 and SALK_082709 accumulated residual levels of miR408, whereas lines SALK_023586 and SALK_038860, hereafter referred to as *mir408-1* and *mir408-2*, respectively, lacked detectable miR408 (Figure 2B). These lines did not show obvious developmental defects when grown *in vitro* on medium supplemented or not with copper (data not shown). The *MIR408* gene was sub-cloned as a 3.3 kbs genomic fragment carrying 1 kb upstream and 1 kb downstream of the pre-miR408 transcript (Figure 3A). Introduction of this fragment into the *mir408-1* null allele restored miR408 accumulation and *Plantacyanine* regulation with the expected Cu response (Figure 3B), indicating that this 3.3 kbs fragment contains all the regulatory elements of the *MIR408* gene. Then, the *MIR408* gene was mutagenized to replace the Adenosine at the 5′ end of the canonical miR408 by an Uridine. The *5′U-MIR408* mutant gene was introduced into the *mir408-1* null allele. Transformants that accumulate 5′A-miR408 and 5′U-miR408 at levels comparable to that of wild-type plants were selected and their progeny was analyzed on the same range of copper concentration. Figure 3B shows that both 5′A-miR408 and 5′U-miR408 are able to down-regulate *Plantacyanin* mRNA levels on copper-free medium, indicating that a 5′Adenosine is not required for miR408-mediated *Plantacyanin* regulation.

## Discussion

AGO1 associates with small RNAs that exhibit a Uridine at their 5′ end, which is the case for most plant miRNAs. Consequently, AGO1 plays an essential role in miRNA-mediated regulation of development and stress responses [3], [23]. It also is essential for siRNA-mediated defense responses against exogenous nucleic acids such as transgenes or viruses. AGO2-associated small RNAs are enriched for molecules that exhibit an Adenosine at their 5′ end [7], [8], [9]. However, until recently, no regulatory function was assigned to AGO2. In fact, AGO2 association was almost considered as a dead end because mutagenesis experiments have revealed that 5′U-miRNAs that normally regulate their targets via AGO1 could no longer regulate their target via AGO2 when transformed into 5′A-miRNAs [7], [12]. However, recent papers have revealed that AGO2 accumulation is induced by viruses and that AGO2 plays a role in antiviral siRNA-mediated defense, sometimes in cooperation with AGO1 [14], [15], [16], . AGO2 is also induced by bacterial pathogens. AGO2 was recently shown to associate with a functional miRNA* molecule that regulates plant immunity along with its cognate miRNA partner. Indeed, miR393, which has a 5′U, associates with AGO1 and targets genes involved in PAMP-triggered immunity, whereas miR393*, which has a 5′A, associates with AGO2 and regulates genes involved in effector-triggered immunity [13].

In this report, miR408 was used as a reporter of AGO2 activity because it carries an Adenosine at its 5′ end and strongly associates with AGO2 in pull-down experiments [8]. Our results strongly suggest redundant roles of AGO1 and AGO2. This result differs from the specialized or redundant roles previously described in the antibacterial or antiviral responses. Indeed, neither *ago1* nor *ago2* single mutations abolish miR408-mediated regulation of *Plantacyanin* mRNA levels, whereas each single mutation has a detectable effect on antiviral or antibacterial responses. Only an *ago1 ago2* double mutant appears compromised in miR408-mediated regulation of *Plantacyanin* mRNA levels, suggesting that AGO1 and AGO2 have redundant roles in this regulation. Moreover, the nature of the 5′ nucleotide of miR408 does not appear essential for its regulatory role because both wildtype *5′A-MIR408* and mutant *5′U-MIR408* genes are able to complement a *mir408* mutant. In addition, the canonical 5′A miR408 molecule associates with both AGO1 and AGO2 [8], [21]. Thus, it is likely that miR408 association with both AGO1 and AGO2 is based on criteria that are not based exclusively on the 5′ end rule. This is reminiscent of miR390 association to AGO7 or miR165/166 association with AGO10 [8], [10]. Indeed, miR390 has a 5′A and as such should associate with AGO2, whereas miR165/166 has a 5′U and as such should associate with AGO1. The fact that miR390 exclusively associates with AGO7 and that miR165/166 almost preferentially associates with AGO10 suggests that the 5′ end rule is a rule used by default when other criteria are not fulfilled. Mutagenesis performed on the *MIR166* gene revealed that the miR166 sequence is not the primary determinant of its predominant association with AGO10, but that specific mispairings and pairings in the miR166/166* duplex determine this association [10]. Similar mutagenesis experiments to express the 5′U miR171, which associates with AGO1, from the *MIR390* precursor stem-loop backbone indicated that the positions of mispairs within the miR390/390* duplex are not sufficient to direct association with AGO7. In addition, mutagenesis experiments to express the 5′A miR390, which associates with AGO7, from the *MIR171* precursor stem-loop backbone indicated that selective association of AGO7 with miR390 cannot be explained by a 5′ nucleotide rule or a foldback-related structure [8]. Further experiments are required to understand the basis of the dual association of miR408 with AGO1 and AGO2.

## Materials and Methods

### Plant material

Seeds from *Arabidopsis thaliana* ecotypes Columbia (Col) were surface-sterilized and plated on culture medium [24] containing 0.8% agar (plant cell culture tested, SIGMA) and range of copper concentration. After 48 hours of incubation at 4°C, plates were transferred to growth chambers under the following conditions: day/night cycles of 16/8 h; light intensity 100–150 lmol m^−2^ sec^−1^, temperature 20°C; humidity 70%.

The *ago1-1* and *ago2-1* mutants have been described before [23], [25]. The *mir408* mutants SALK_023586, SALK_038860, SALK_081087 and SALK_082709 were isolated from the Salk Institute collection of T-DNA mutants, and genotyped using forward (5′- ATGACAGAGAGGTAGACCAAACCC -3′) and reverse (5′- CAGAACCCTCCAGCTAATTTAGAGGG -3′) primers specific to the *MIR408* gene, and T-DNA primer LBa1 (5′-ATGGTTCACGTAGTGGGCCATC-3′).

### Cloning and mutagenesis

The MIR408 genomic region was amplified as a 3.3 kbs fragment using MIR408-fwd (5′ – GCGCGCTCTAGATGTATAATGACAGGAATGGAACCTC– 3′) and MIR408-rev (5′ – GCGCGCCTCGAGCCAGAGTTCACCGTACGGAC– 3′) primers and cloned in pGMT easy (PROMEGA). Following digestion with restriction enzymes corresponding to the underlined sequences, the MIR408 fragment was cloned into pGREENII [26]. Once sequenced and validated by transient expression in benthamiana for miR408 production, this plasmid was mutagenized to change miR408 5′ base from an A to a U. Plasmid amplification was done using pFU turbo (STRATAGENE) and primers 5′ – GAAGAGGCAGTGCAAGGGTAGAGACAAAAC – 3′ and 5′ – GTTTTGTCTCTACCCTTGCACTGCCTCTTC– 3′ (mutagenised base is underlined). Amplified DNA was digested with DpnI and transferred to E. coli. Mutagenized clones were sequenced before transfer to Agrobacterium.

### RNA analysis

Total RNA was isolated from whole plant grown in vitro, from 2 days post germination (dpg) to 15 dpg. Small RNA were separated on denaturing 15% polyacrylamide gels, followed by blotting to a nylon membrane (Genescreen Plus, PerkinElmer Inc) as described before [27]. Blots hybridization was performed using gamma-ATP ^32^P end-labeled oligonucleotides as described before [27]. A labeled oligonucleotide complementary to U6 was used for normalisation. For high molecular weight gel blot analysis, a 1% agarose denaturing gel was used to separate RNA. Transfer was then performed using a nylon membrane (Genescreen Plus, PerkinElmer Inc). Blots were hybridized with an alpha-dCTP ^32^P labeled DNA probe complementary to Plantacyanin mRNA in Church buffer [28]. Plantacyanin probes were generated by PCR using primers *5′-* GATCAATGGCCAAGGGAAGAGGCAG *-3′* , *5′-* CGATCAAACCGCGGTGACTGCG *-3′* followed by random priming (Prime-a-Gene® Labeling System, PROMEGA).
